# Fauna used by rural communities surrounding the protected area of Chapada do Araripe, Brazil

**DOI:** 10.1186/s13002-016-0115-x

**Published:** 2016-09-19

**Authors:** Kallyne Machado Bonifácio, Alexandre Schiavetti, Eliza Maria Xavier Freire

**Affiliations:** 1 Centro de Biociências, Curso de Doutorado em Desenvolvimento e Meio Ambiente, Laboratório de Herpetologia, Universidade Federal do Rio Grande do Norte/UFRN, Natal, RN CEP59078-900 Brasil; 2 Departamento de Ciências Agrárias e Ambientais, Universidade Estadual de Santa Cruz/UESC, Km 16 Rodovia Ilhéus-Itabuna, Ilhéus, BA CEP45662-000 Brasil; 3 Centro de Biociências, Departamento de Botânica e Zoologia, Universidade Federal do Rio Grande do Norte/UFRN, Natal, RN CEP59078-900 Brasil

**Keywords:** Wild animals, Intracultural variation of knowledge, National Forest

## Abstract

**Background:**

Studies on the inter-relations between people and animals have been considered essential to better understand the dynamics of socio-ecological systems. This study aimed to register the animal species known by the communities adjacent to National Forest of Araripe, their uses and if the close relationship affects the knowledge of useful species.

**Methods:**

Data collection was conducted through a semi-structured inquiry form, free listings and guided tour. The study included 246 people from two community groups: group 1 (*n* = 113; <2 km from FLONA) and group 2 (*n* = 133; ≥ 2 km).

**Results:**

According to the free listing, group 1 communities know more animal species (11.50 ± 5.81) than group 2 (9.41 ± 3.70), with a significant difference in knowledge between the groups. Men and women showed no significant difference in knowledge about animal species. The men from group 1 know, significantly, more species than men from group 2; but this difference was not observed in women from both groups. In the analysis of the Use Value (UV), *Mazama gouazoubira* showed a higher UV, both in group 1 (1.15) and group 2 (1.49). The guided tour identified the presence of 11 species, common in the vegetation of Forested Savannah (*Cerradão*) and in the transition Rainforest/Savannah (Cerrado).

**Conclusion:**

The results indicate *M. gouazoubira* as the most known and used species in this Protected Area, showing that species of interest to the local communities are worthy of conservation attention.

## Background

In ethnozoology, the fauna inventory has been used to provide information on the repertoire of animal species most used for varied purposes [1, 2] such as feeding [3] medicinal [4], pets [5], and magic-religious [6], bringing attention to conservation aspects related to these uses. For example, a study based on analysis of the hunting activities by communities located in different areas of Ceará, in northeastern Brazil, documented 27 species of reptiles with some utilitarian value, of which five are listed as endangered species in the Brazilian list. These results emphasize the importance of these areas for the maintenance of the herpetofauna [3].

Recent studies highlights that anthropogenic pressures on forest areas, combined with the versatility of uses and illegal trade of wildlife, tend to intensify the population decline of animal species in Brazil [7–10] and in different countries as Argentina [11], Mexico [12] and Spain [13]. In the Brazilian Amazon, a study of mammal hunting in indigenous and rural groups found that the lack of knowledge of local people about the importance of some animal species to the environment also contributed to this decline [14]. Thus, for decision-making regarding the priority sites for conservation and management, it is essential to determine which species are locally important, as well as their preferred habitats in the landscape matrix, because these analyses will point out ecological aspects and threat factors for the protection and conservation of these species.

Worldwide speaking, one of the difficulties faced by managers of protected areas in handling biological species is the lack of information about their composition and environments they use [15]. The National Forest (FLONA, VI IUCN category) of Araripe, although highlighted as an area of extreme biological importance for the conservation of the fauna of the *Caatinga* biome [16], lacks further studies on its fauna, limiting management and conservation actions. Past studies are only surveys on species in different vegetation types, such as birds [17], entomofauna [18], herpetofauna [19, 20] and mammals [21]. With a focus on ethnozoology, studies were developed within ornithology, with emphasis on composition and traditional use of birds [22] and mammals [23]. However, there are no published data that addresses the richness of the fauna of this protected area, integrated with local knowledge and ecological analysis.

Given the above, this study aimed to collect data on the animal species known to local communities, as well as their uses and places of occurrence in the vicinity of the National Forest of Araripe, Ceará, taking into account the effects of these variables: the distance between the communities and the FLONA, and the gender regarding knowledge on the richness of known animals. We also aimed to propose priority sites for conservation. Specifically, we intended to answer the following questions: what are the known species reported as utilitarian for local communities? Which species have higher use value, deserving more attention in conservation policies? Which phytophysiognomies are potential for conservation of animal species in the area studied?

## Methods

### Area of study

**Fig. 1:**
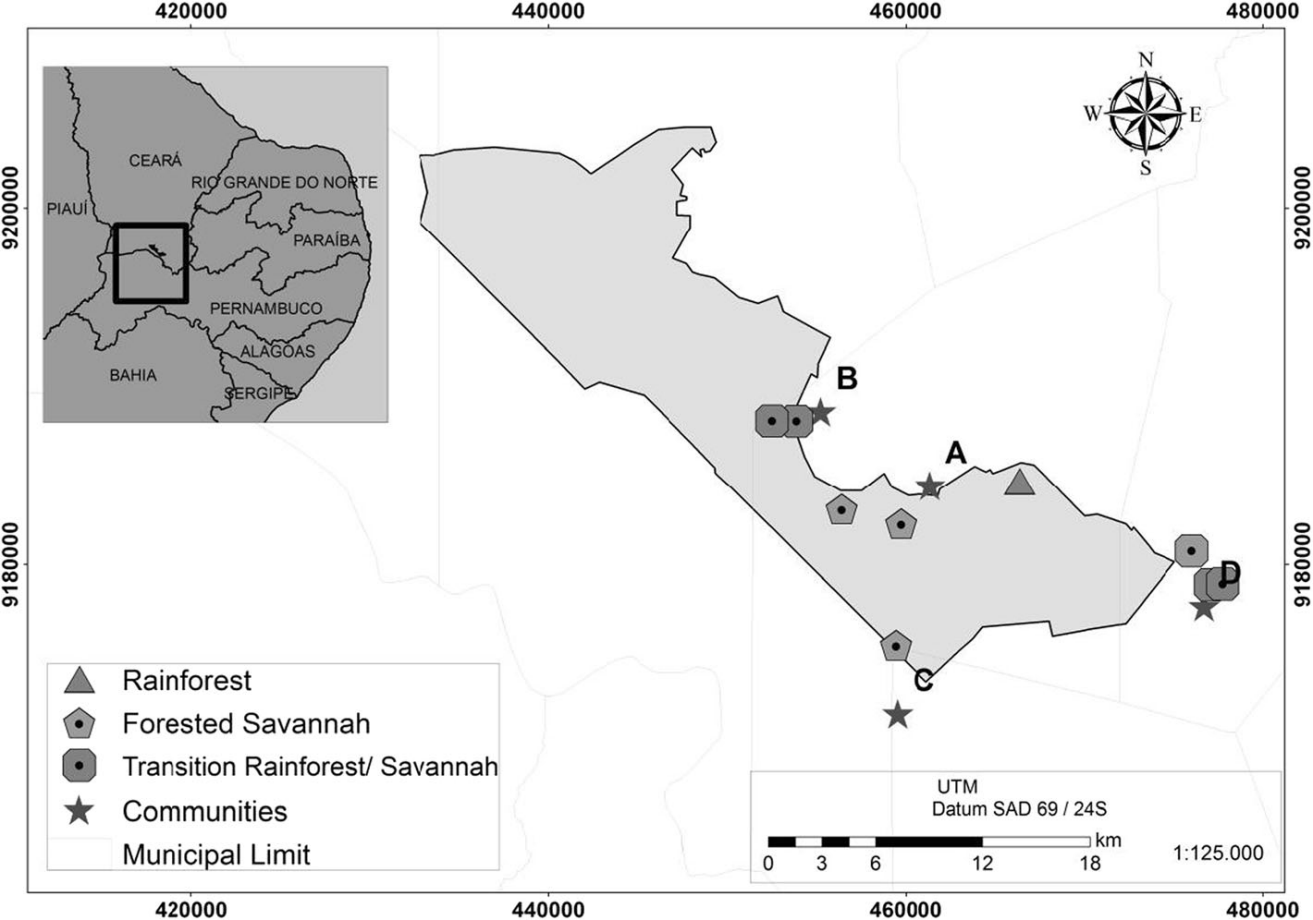
FLONA of Araripe, communities: A- Caldas, B- Farias, C- Novo Horizonte and D- Banco de Areia, Ceará, Brazil

This study was conducted in four communities located in the buffer zone of the National Forest of Araripe, Ceará, a semiarid region of Northeast Brazil, with two located near this protected area (< 2 km; Group 1) and two being more distant (≥2 km; Group 2), respectively (Fig. 1): Caldas and Farias communities (belonging to the municipality of Barbalha), Novo Horizonte community (municipality of Jardim) and Banco de Areia community (municipality of Missão Velha).

Founded in 1946, the FLONA of Araripe (Category VI IUCN) is the first Protected Area for the Sustainable Use of resources established in Brazil and currently stands out as an area of extreme importance for the conservation of flora and fauna of the *Caatinga* biome [16]. With a land area of 38,919.47 hectares, it covers 05 municipalities in the State of Ceará (Northeast region of Brazil) [24]. It comes under the influence of hot and humid tropical climate, annual average rainfall of 1090.9 mm, temperatures around 23 °C and topography with altitude reaching 900 m, characterized by flat terrain features and sedimentary lithology [25, 26].

According to the regional classification of vegetation, the studied communities are distributed in three forest types (Fig. 1): Rainforest (Caldas community), Forested Savannah “*Cerradão”* (Novo Horizonte community) and Rainforest *“Mata Úmida”*/Savannah transition “*Cerrado”* (Farias and Banco de Areia communities) [27]. Briefly, the rainforest vegetation is characterized by high density and high-sized trees (heights over 15 m); the forest savannah is characterized by a reduction in the size of the trees, forming a dense, closed vegetation, and the savannah is characterized as an open formation with ununiformed and twisted trees, distant from each other [28]. These types of vegetation include forest fragments in different stages of conservation, which are used for different purposes as a path between local communities, wild resources extraction (of pequi, *Caryocar brasiliense* Camb. and *fava d’anta*, *Dimorphadra gardneriana* Tul.) and subsistence agriculture (corn, beans, and cassava) [26].

Regarding the fauna, it is home to two species of birds listed as endangered: the araripe manakin *Antilophia bokermanni* (Coelho & Silva, 1998), the only bird with occurrence restricted to the state of Ceará [29], and the araponga-of-the-northeast *Procnias averano averano* (Hermann, 1783) [30].

### Ethnozoological data collection

The ethnozoological data was collected for three consecutive months (January to March, 2013) through interviews, using a semi-structured inquiry form [31], directed to residents with a minimum age of 18 years and residence time on site for at least 10 years. Only one member of each family was interviewed, selected through the Snowball technique [32], which consists of selecting survey participants from recommendation. The form addressed issues regarding animal species of hunting interest for locals and their known uses, as well as socio-economic data for each participant, such as gender, age, residence time on site and occupation.

The study included 246 people (173 men and 73 women), of a total of 615 families estimated by health workers in each community, with 113 people distributed in a radius of <2 km from the FLONA and 133 within the radius of ≥2 km; overall average age was 54 years old (Table 1).

**Table 1:** General data of the studied community groups surrounding the FLONA of Araripe, Ceará, Brazil

Characteristics	Group 1 (< 2 km)	Group 2 (≥ 2 km)
Number of interviewees (frequency in %)	81 M (71.68 %)	92 M (69.17 %)
	32 W (28.31 %)	41 W (30.82 %)
Age (mean and standard deviation)	50.2 ± 18.20	57.23 ± 17.55
Time of residence (years – means and standard deviation)	44.98 ± 18.58	52.03 ± 19.7
Main occupation (frequency in %)	Agriculturist (23 %)	Agriculturist (39.85 %)
	Retired (40.7 %)	Retired (18.79 %)

*M* men, *W* women

To gather information regarding the perceived richness of animal species and their uses, we employed free listing [31], a process in which the participant was asked to mention the species they knew. The free listing technique is to ask each participant to the question of research interest, in order to evaluate responses patterns. In this technique it is assumed that the culturally most important elements appear in the lists in order of importance [31]. An album containing illustrative figures of animal species occurring in the region, prepared based on the list of species documented in the Management Plan of the National Forest of Araripe [25], was presented to each of the participants, to help them identify the species mentioned in the free listings.

We also used guided tour [33], as a method to confirm the occurrence of these species in the area of study, having been made eight field trips on consecutive days, 5 h duration each. This method consisted in visiting areas of the National Forest and its surroundings, in the company of community of people with knowledge of the local fauna, looking (at random) for trace elements (footprints or carcasses) to identify the animals mentioned. They walked through forest fragments (caponier, dense forest, shore paths), through pre-existing trails often used by hunters in hunting or extractive activities, as well as the likely occurrence of the species locations, based on the experience of the informant. The forest fragments sampled (*n* = 9) were located in the Rainforest vegetation (*n* = 1), the Forested Savannah (*n* = 3) and the transition Rainforest/Savannah (*n* = 5) (Fig. 1). The sampling effort in the studied fragments can be seen in Table 2.

**Table 2:** Effort made in the nine trails/roads undertaken in each type of vegetation of the FLONA of Araripe, Ceará, Brazil

Vegetation physiognomy	Geographic location (UTM)	Time/ Men (T/M)	Distance travelled (km)	Effective effort (km. H/H)
Rainforest	0466382 / 9184682	30	14	3.88
Forested Savannah	0460333 / 9183172	30	42	11.66
	0459441 / 9175524			
	0456400 / 9183120			
Transition Rainforest/Savannah	0454877 / 9188692	30	52	14.44
	0453881 / 9188058			
	0452518 / 9188078			
	0477096 / 9178872			
	0477750 / 9178922			

The scientific nomenclature used for the species mentioned in this study followed the standards set by the Brazilian Herpetological Society [34], by the Brazilian Committee of Ornithological Registration [35], and the Annotated list of Mammals of Brazil [36].

The threat status of each species was obtained in the National List of Brazilian Endangered Fauna Species [37] and in the International List Endangered Species of the International Union for Conservation of Nature—IUCN [38]

### Data analysis

#### Sampling effort and species perceived wealth

The relative sampling efficiency regarding the number of interviews conducted in community groups (Groups 1 and 2) was evaluated by cumulative curves of species [39], constructed with data regarding the presence and absence of animal species mentioned in the interviews (one interview = one sample). We used the nonparametric estimator Chao 2, which is based on the wealth of species found in one sample and found in exactly two, requiring only the use of an array of qualitative data (presence or absence) [40]. For these characteristics, the Chao 2 is indicated for ethnozoological studies [41]. Curves were generated using the EstimateS 8.2.0 program with 1000 randomizations and 95 % confidence interval [42].

Wealth of species was estimated by traces elements (footprints or carcasses). To quantify the effect of individual records, we considered that different tracks of the same species in the same trail/road would be different records; for this, we used as a parameter, the direction followed by the animal and/or size of the footprint [43]. The footprints were identified by the informant himself and then photographed; carcasses were identified with the help of experts.

#### Calculating the use value

The local relative importance of known animal species was determined by calculating the commonly use value (UV) and effected by the formula; *UV* = ∑ *U*/*n*; in which, UV = index of the species use value; U = number of citation by etnospecies; *n* = number of participants [44]. Use categories were determined according to the literature [45]: feeding, medicinal, mystic-religious, handcraft, ornamental and other uses, making no distinction between the current and potential uses. For animals cited for therapeutic indications, conditions were grouped into categories according to the International Statistical Classification of Diseases and Related Health Problems—ICD-10 [46]. Additionally, we analyzed the citations of more representative uses for calculating Fidelity Level (FL) obtained by: FL = Ip/Iu x 100 %; in which, FL = fidelity level; Ip = number of informants, who suggest the use of a particular animal to a principal use and Iu = total number of participants, who cited the animal for any purpose [47]. According to this index, the greater the consensus among participants regarding the uses mentioned for a species, the higher the FL. The FL may exhibit values equal to 100 % [48].

For complimenting data, the Mann-Whitney test, 5 % significance level, was used to verify the existence of differences in the richness of known animals by gender and distance of local communities to the Protected Area. We analyzed data with the Software SPSS 20.

## Results and discussion

### Perceived richness of animal species

We registered a total of 53 species cited by participants. Group 1 communities reported a greater number of species (52), belonging to 50 genera and 31 families, with an average citation of 11.50 ± 5.81 per participant. Participants from Group 2 mentioned 44 species, from 41 genera and 25 families, with an average citation of 9.41 ± 3.70 (Table 3). These differences in the richness of species and number of citations are significant (Mann-Whitney U = 6128, *p* = 0.012), leading to the conclusion that the spatial distribution of human populations in relation to a natural area can contribute to a greater or lesser local knowledge on the fauna with utilitarian value or not.

**Table 3:** Animal species cited by surrounding community Groups of the FLONA of Araripe, Ceará, Brazil

Family/ Species	Local Name/N° of citations	Conservation Status	
		MMA	IUCN
BIRDS			
Tinamidae			
*Crypturellus noctivagus zabele* (Spix, 1825)	“zabelê” (yellow-legged tinamou)/21	VU	NT
*Crypturellus parvirostris* (Wagler,1827)	“nambu” (Small-billed tinamou)/73	─	LC
*Nothura maculosa* (Temminck, 1815)	“corduniz” (spotted nothura)/65	─	LC
Accipritidae			
*Rupornis magnirotris* (Gmelin, 1788)	“gavião” (hawk)/23	─	LC
Falconidae			
*Caracara plancus* (Miller, 1777)	“carcará” (southern carcara)/18	─	─
Cracidae			
*Penelope superciliaris* (Temminck, 1815)	“jacu” (guan)/211	─	LC
Carimidae			
*Cariama cristata* (Linnaeus, 1766)	Sariema/102	─	LC
Columbidae			
*Columbina minuta* (Linnaeus, 1766)	“rolinha comum”(common turtle dove)/48	─	LC
*Columbina talpacoti* (Temminck, 1811)	“rolinha caldo de feijão” (ruddy ground dove)/17	─	LC
*Columbina squamatta* (Lesson, 1831)	“rolinha cascaval” (scaled dove)/17	─	─
*Leptotila verreauxi* Bonaparte, 1855	“juriti” (white-tipped dove)/70	─	LC
*Zenaida auriculata* (Des Murs, 1847)	“ribaçã” (eared dove)/10	─	LC
Psittacidae			
*Eupsittula cactorum* (Kuhl, 1820)	“guinguirro” (cactus parakeet)/24	─	LC
*Forpus xanthopterygius* (Spix, 1824)	“pacu” (Blue-winged parrotlet)/6	─	─
Cuculidae			
*Crotophaga ani* Linnaeus, 1758	“anu preto” (smooth-billed ani)/2	─	─
*Guira guira* (Gmelin, 1788)	“anu branco” (guira cuckoo)/1	─	LC
*Piaya cayana* (Linnaeus, 1766)	“alma de gato” (squirrel cuckoo)/7	─	LC
Strigidae			
*Glaucidium brasilianum* (Gmelin, 1788)	“caburé” ferruginous pygmy owl)/12	─	LC
*Megascops choliba choliba* (Vieillot, 1817)	“coruja” (owl)/12	─	LC
Nyctibiida			
*Nyctibus griséus* (Gmelin, 1789)	“mãe da lua”/12	─	─
Trochilidae			
*Chlorostilbon luciduss* (Shaw, 1812)	“bizunga”/12	─	─
*Eupetomena macroura* (Gmelin, 1788)	“tesourão” (swallow-tailed hummingbird)/2	─	LC
Bucconidae			
*Nystalus maculatus* (Gmelin, 1788)	“fura-barreiro” (puffbird)/2	─	LC
Picidae			
*Veniliornis passerinus* (Linnaeus, 1766)	“pica-pau-pequeno” (woodpecker)/14	─	─
Tyrannidae			
*Fluvicola negenta* (Linnaeus, 1766)	“lavadeira”/5	─	─
*Pitangus sulphuratus* (Linneus, 1766)	“bem-te-vi” (great kiskadee)	─	LC
Pripidae			
*Antilophia bokermanni* Coelho & Silva, 1998	“soldadinho-do-araripe” (Araripe manakin)/20	CR	CR
Corvidae			
*Cyanocorax cyanopogon* (Wied, 1821)	“cancão” (white-naped jay)/33	─	LC
Troglodytidae			
*Pheugopedius genibarbis* (Swainson, 1838)	“chorró” (moustached wren)/6	─	LC
Turdidae			
*Turdus leucomelas* Vieillot, 1818	“sabiá comum” (pale-breasted thrush)/34	─	LC
*Turdus rufiventris* (Vieillot, 1818)	“sabiá peito amarelo” (rufous-bellied thrush)/13	─	LC
Thraupidae			
*Sicalis flaveola* (Linnaeus, 1776)	“canário da terra” (saffron finch)/8	─	LC
Parulidae			
*Setophaga fusca* (Statius Muller, 1776)	“papo de fogo” (lackburnian warbler)/3	─	LC
*Myiothlypis flaveola* Baird, 1865	“canário comum” (common canary)/9	─	LC
MAMMALIA			
Didelphidae			
*Didelphis albiventris*(Lund, 1840)	“cassaco” (white-eared Opossum)/27	─	LC
Dasypodidae			
*Cabassous unicinctus* (Linnaeus, 1758)	“china” (southern naked-tailed armadillo)/69	─	LC
*Dasypus novencimctus* (Linnaeus, 1758)	“tatu comum” (common armadillo)/195	─	LC
*Euphractus sexcinctus* (Linnaeus, 1758)	“peba” (six-banded armadillo)/194	─	LC
Canidae			
*Cerdocyon thous* (Linnaeus, 1766)	“raposa” (fox)/82	─	LC
*Nasua Nasua* (Linnaeus, 1766)	“guará” (South American coati) /20	─	LC
*Procyon cancrivorus* (G. Cuvier, 1798)	“guaxinim” (raccoon)/19	─	LC
Mustelidae			
*Conepatus semistriatus* (Boddaert, 1785)	“gambá” (striped hog-nosed skunk)/51	─	LC
*Galictis vitatta* (Schreber, 1776)	“furão” (ferret)/15	─	LC
Felidae			
*Leopardus tigrinus* Shreber, 1775	“gato do mato; lagartcheiro” (oncilla; tiger cat)/45	EN	VU
*Leopardus wiedii* (Schinz, 1821)	“gato maracajá” (margay cat)/7	VU	─
*Panthera onca* Linnaeus, 1758	“onça pintada” (spotted jaguar)/45	VU	NT
*Puma concolor (*Linnaeus, 1771)	“onça vermelha” (red jaguar)/130	VU	LC
*Mazama gouazoubira* (G. Fischer, 1814)	“veado comum” (common deer)/237	─	LC
Caviida			
*Galea spixii* (Wagler, 1831)	“preá” (guinea pig)/40	─	LC
Dasyproctidae			
*Dasyprocta prymnolopha* (Wagler, 1831)	“cutia” (Black-rumped agouti)/205	─	LC
Myrmecophagidae			
*Tamandua tetradactyla* (Linnaeus, 1758)	“tamanduá” (Collared anteater)/113	─	LC
REPTILIA			
Iguanidae			
*Iguana iguana* (Linnaeus, 1758)	“camaleão” (chameleon)/38	─	─
Teiidae			
*Salvator merianae* (Duméril &Bibron,1839)	“teiú” (black and white tegu”)/88	─	LC

Legend: Categories of the Red List of IUCN (2014.1): *DD* Data deficient, *LC* Least Concern, *NT* Near Threatened, *VU* Vulnerable, *CR* Critically endangered. Categories of Brazilian Red List (MMA, 2014): *CE* Critically endangered, *E* Endangered, *VU* Vulnerable

**Fig. 2:**
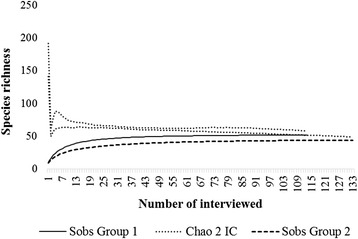
Cumulative curve of mentioned species. Group 1: 52; Expected richness: 52.33; Grupo 2: 44; Expected richness: 44.25. CI: confidence interval of 95 %

The number of interviews (*n* = 246) showed a satisfactory sample of the species richness locally known by participants from Group 1 and Group 2, considering that the richness recorded was of 99.6 % (*n* = 52) and 99.4 % (*n* = 44), respectively, of the total species estimated by Chao 2 for these areas (Fig. 2).

The group of Birds was the most often cited (n_Group 1_ = 33 species and n_Group 2_ = 25), followed by Mammals (n_Group 1_ = 17 and n_Group 2_ = 17) and Reptiles (n_Grupo 1_ = 2 and n_Grupo 2_ = 2). Birds and mammals are typically the most well represented groups in studies that address the knowledge and use of wild vertebrates by human populations, referred to as the zoological groups most relevant to local communities [41, 49–52].

Among the species cited by participants of the Group 1 communities, stand out in the number of citations: *Mazama gouazoubira* (G. Fischer, 1814) (Common deer, 103 citations), *Penelope superciliaris* (Temminck, 1815) (Rusty-margined guan; 98) *Dasypus novemcinctus* (Linnaeus, 1758) (Nine-banded armadillo; 95) and *Euphractus sexcinctus* (Linnaeus, 1758) (Six-banded armadillo; 94). On the other hand, communities of Group 2 cited more: *M. gouazoubira* (Common deer, 129 citations), *P. superciliaris* (Rusty-margined guan; 110); *E. sexcinctus* (Six-banded armadillo; 101), *D. novemcinctus* (Linnaeus, 1758) (Nine-banded armadillo, 98) and *Dasyprocta prymnolopha* (Wagler, 1831) (Black-rumped agouti; 99). These species were greatly popular as a food resource in different locations in Brazil [41, 50, 53, 54] and Mexico, signaling a standard regarding the animal species most important for hunting [12, 55, 56]. The predominance of citations for the common deer, *M. gouazoubira*, in both groups of communities studied, suggests that the species is possibly distributed in different forest fragments frequented by human populations, allowing the common deer to be more vivid in the memory of these people. The prominence of a species results from the degree of interaction of people with this species, which may be due to its abundance in area [57].

Comparing the number of species cited by men and women in the communities studied, on average men cited more species than women (men = 10.35 ± 4.88 animals; women = 10.24 ± 4.67), with such differences as statistically significant by the Mann-Whitney test (U = 6811, *p* = 0.697). Analyzing men and women separately, we found that the men from Group 1 communities cited more animals than men from Group 2 communities (on average 11.50 ± 5.81 and 9.41 ± 3.69 species, respectively; Mann-Whitney U = 3025, *p* = 0.033), though this difference did not exist among women of the studied groups (Mann-Whitney U = 550, *p* = 0.237). This occurs, because men and women have different and unique relationships with biodiversity. The propensity of men to be in possession of greater knowledge about wild animals in this study was similar to that found in the semiarid region of the state of Paraíba [41]. This can be explained due to men participating in activities that provide a more direct interaction with the environment, such as hunting, gathering and farming [58, 59]. The fact that Group 1 men mentioned a greater number of animals compared to men from Group 2, may be due to the life experience of those people with the local wildlife, which includes access to diversity of knowledge about wildlife resources at different stages of life and the use of the natural environment, according to the practiced activity, since differences in the knowledge of communities regarding biological species may be closely associated with the productive activities of human populations [55].

Ethnobiological studies focusing on intracultural variation of knowledge of wildlife have shown that, besides the distance of local communities in relation to the environment, socio-cultural variables such as gender, age classes, schooling, housing time, type of practiced activity and income all tend to influence the knowledge of local resources by human populations [41, 60–62].

### Species richness by sampling in forest fragments and ecological aspects

The sampling method by trace elements, through footprints (72 footprints of 10 species, 95.8 %) and carcasses (*n* = 1 sp.; 4.2 %) confirmed the presence of 11 species (seven mammals, three birds and one reptile) to 11 genera and 10 families (Table 4). The greater occurrence of species was found in the Forested Savannah vegetation (8 spp.; 72.72 % of total records), followed by the transition area Rainforest/Savannah (6 spp.; 54.55 %) and Rainforest (3 spp.; 27.27 %). Mammal species accounted for 63.64 % (*n* = 7 spp.) of the total representation in sampled areas, followed by birds (27.27 %; 3 spp.) and reptiles (9.9 %; 1 spp.). According to the Geoenvironmental Zoning of the state of Ceará, the Savannah is one of the vegetation units that has the best storage conditions [28]. Its closed environment (“capões forest”) and dense configuration provides favorable environmental conditions to meet the needs of animal species that include availability of food, rest, refuge, and shelter.

**Table 4:** Animal species recorded in forest fragments sampled by the walke-in-the-woods in the FLONA of Araripe, Ceará, Brazil

Family/ Specie/ Local name	Vegetation types / Number of records			Records Type	Total
	Rainforest	Forested Savannah	Transition Rainforest/Savannah		
BIRDS					
Falconidae					
*Caracara plancus (*Miller, 1777) southern carcara/“carcará”	…	…	1	footprints	1
Cracidae					
*Penelope superciliaris* (Temminck, 1815) Rusty-margined guan/“jacu”	…	3	5	footprints	8
Strigidae					
*Megascops choliba (*Vieillot, 1817) Owl/ “coruja”	…	1	…	footprints	1
MAMMALIA					
Canidae					
*Cerdocyon thous (*Linnaeus, 1766) Fox/“raposa”	…	2	…	footprints	2
*Nasua Nasua* (Linnaeus, 1766) South American coati/“guará”	…	1	…	footprints	1
Mustelidae					
*Conepatus semistriatus* (Boddaert, 1785) Striped hog-nosed skunk/“gambá”	…	1	…	footprints	1
Felidae					
*Leopardus tigrinus* (Shreber, 1775 Oncilla/“gato do mato”	…	…	1	footprints	1
Cervidae					
*Mazama gouazoubira* (G. Fischer, 1814) Common deer/“veado comum”	…	6	17	footprints	23
Dasyproctidae					
*Dasyprocta prymnolopha* (Wagler, 1831) Black-rumped agouti/“cutia”	1	9	9	footprints	19
Myrmecophagidae					
*Tamandua tetradactyla* (Linnaeus, 1758) Collared anteater/“tamanduá”	3	…	…	carcasses	3
REPTILIA					
Teiidae					
*Salvator merianae* (Duméril & Bibron,1839) Tegu/“teiú”	1	2	7	footprints	10
TOTAL	5	27	40	…	72

The fragments sampled in the Rainforest vegetation correspond to environments predominantly open (such as trails within the forest and road) that, by the degree of disturbance, becomes an interfering factor for the presence of species in this site. This finding was corroborated by Negrão and Valladares-Pádua [63] in a Forest Reserve of São Paulo, Brazil, to find that the traffic of people and vehicles through the Reserve may have contributed to the absence of the Spotted (*Agouti paca* Linnaeus, 1766) in the region, which is sensitive to the presence of humans.

The higher frequencies of the recordings were made by the common deer, *M. gouazoubira* (*n* = 23; 31.94 % of total records), especially in transitional vegetation Rainforest/Savannanh, and the Black-rumped agouti, *D. prymnolopha* (*n* = 19; 26.38 %). Of all the recordings (*n* = 72), only the Black-rumped agouti, *D. prymnolopha* (*n* = 19; 26.38 %) and the tegu, *Salvator merianae* (Duméril & Bibron, 1839) (*n* = 9; 12.5 %) were recorder in all three vegetation types: Forested Savannah (9.02 records), transition area Rainforest/Savannah (9 and 7, respectively) and Rainforest (1 record each).

The most frequent records for *M. gouazoubira* (Common deer) and *D. prymnolopha* (Black-rumped agouti) may be related to territorial behavior and/or feeding strategy of these species, which are influenced by environmental characteristics of forest fragments. As pointed out by Caraballo [64], animal species elect different habitats according to their annual or seasonal needs.

The *M. gouazoubira* species (common deer) occurs in various environments, from continuous dense forests to open savannahs with small and few forest patches, but always associated with forests for shelter and food [65]. About the diversity and geographical distribution of terrestrial, medium and large mammals in the northeastern Brazil, Feijó and Langguth [66] mention the current presence of *M. gouazoubira* within the state of Ceará, but restricted mainly to the most conserved regions of the mountain area, where access is difficult.

*D. prymnolopha* (Black-rumped agouti) is a species of rodent that feeds on a variety of fruits and seeds in Neotropical forests and savannahs [67], also consuming leaves, flowers and fibers [68]. Feijó and Langguth [66] comment that agoutis have high vagility and have also suffered translocations of anthropogenic origin. These factors may contribute to the presence of this animal in several forest fragments, which was confirmed in this study by the evidence of its presence in forest fragments of the three sampled vegetation types.

The reptile fauna of the Chapada of Araripe is represented predominantly by species that are typical of the *Caatinga*, with occurrences in the areas of Savannah and Rainforest [19]. The only recorded species was the *S. merianae* lizard (tegu), which occurs in a variety of habitats (Savannah, Caatinga and Rainforest) [69] and in semi-arid \regions of Brazil. It is the most hunted reptile [70]. Omnivorous in habit and quite tolerant to more open environments, the species lives at the edges and clearings of the forest, often approaching anthropogenic environments [71, 72]. According to Zanella [73], the *S. merianae* has great ability to shift between different types of environments, which may have contributed to the recording of this species in the three sampled vegetation types. We hypothesize that the unusual weather conditions during the course of the field study (year of drought and prolonged drought in the state of Ceará), possibly motivated a higher frequency of movement of this lizard between environments, seeking for food. This is in line with Silva [74] in his study of diet and reproductive aspects of the *S. merianae* in an adjacent community of the FLONA of Araripe, Ceará. These authors found that in the warmer months of the year (from October to February) the tegu, *S. merianae*, showed a greater recording of location changes for reproduction events, and certainly for foraging, since it is a period of less availability of food resources.

We observed during the period of study, in the Rainforest area (*n* = 3 records) using trampling records, the occurrence of the Collared anteater, *Tamandua tetradactyla* (Linnaeus, 1758) in the FLONA of Araripe. The three specimens were found in the same stretch of road (open green area), which may be an indication that *T. tetradactyla* density is high in this forest fragment. This species has a wide distribution in the state of Ceará, being found in different vegetation types that include areas of open vegetation and dense forest [66]. With daytime, twilight and nocturnal habits, the Collared anteater, *T. tetradactyla*, has a low metabolism, changing the pattern of activity according to the change in environment temperature, which leaves it more vulnerable to the risk of accidents [75].

Regarding the conservation status of the species recorded through interviews and confirmed by the trace elements method, only the Oncilla *Leopardus tigrinus* (Shreber, 1775), is listed in the National List of Brazilian Fauna Endangered Species and is considered endangered (EN) [76]. In the Official List of Endangered Species of the IUCN, the *L. tigrinus* species (Shreber, 1775) is assessed as Vulnerable. The other species are classified as Less Concerning [77].

### Animal species of utility value

After the inclusion of species in utility categories, 15 species (distributed in 11 families and 14 genera) presented some known use, corresponding to 28.3 % of all species mentioned. These were grouped into six categories of use: feeding, medicinal, handcraft, ornamental, mystic-religious and other uses (Table 5).

**Table 5:** Number of animal species by category and type of use cited by community groups of the FLONA of Araripe, Ceará, Brazil

Category of uses	Types of known uses	No. of cited species	
		Group 1	Group 2
Food resource	human food	10	10
Handcraft (for the leather)	cooking appliances (blanket, knapsack, gibbon, belt); making of musical instruments (tambourine); furniture (upholstery and stool)	1	2
Medicinal	ear diseases (ear pain, deafness); respiratory diseases (cough, sore throat, asthma); digestive system diseases (toothache); musculoskeletal diseases (rheumatism, back pain), eye disease (conjunctivitis), diseases of the circulatory system (hemorrhoids), poisoning (snake bite); symptoms, signs and abnormal clinical examinations (cracks in the feet, mycosis)	5	2
Ornamental	wall hangings (room decoration)	0	1
Mystic	making of keychains	0	1
Other uses	making tools (needle for sewing leather); harness; sheath for knife	1	3

In the use analysis by group of communities, Group 1 participants attributed uses for 15 species belonging to 10 families. Among participants from Group 2, 14 species were cited, all mentioned by participants from Group 1. The feeding (10 species cited by participants from both groups of communities) and medicinal categories (5 and 7 species cited by participants in Groups 1 and 2, respectively) united a greater number of species known to be locally useful. The categories that had the greatest number of useful species are also the most representative in the Brazilian semi-arid regions [6, 53] and other biomes of Brazil, such as the Atlantic Forest [49, 50, 52], demonstrating the cultural relevance attributed to these uses. Barboza et al. [78] studying hunting activities for meat in different parts of the Caatinga biome, found that the mammals’ meat are the main source of protein for human communities of Brazilian semi-arid. The use of wildlife species as food and as medicinal resources for local communities is still a common practice in Brazil [79] and in other countries, particularly in arid environments [80], where poverty finds in the flesh of these animals and its by-products, energetic benefits and an alternative for the treatment of different diseases.

The average use value among Group 1 participants was 0.40 and for the Group 2, 0.42 (Table 6). The common deer, *M. gouazoubira* had higher use values with 1.15 to participants from Group 1 (131 citations; FL = 88.67 %) and 1.49 to participants from Group 2 (199 citations; FL = 67.93 %); the guan, *P. superciliaris*, 0.87 to participants from Group 1 (98 citations; LF = 100 %) and 0.85 to participants from Group 2 (114 citations; LF = 99.11 %). There was no significant difference between studied Groups regarding the use value assigned to the list of known animal species (U = 30, Z = -6302, *p* > 0.05), indicating that community groups have similar values for the same species. According to Medeiros and Albuquerque [81], the environment can bring together or distance communities in terms of similarity in actual and/or cognitive use of biological resources. Animal species highlighted by their usefulness are well distributed in forest fragments of the FLONA of Araripe, not only that, the history of local use of the studied groups is the same, which may explain the similarity in the uses given to animals in the area of study. As suggested by Medeiros [82], who highlighted the factors (cultural and ecological) shared by different human populations as a possible explanation for certain similarities in the use of a biological resource.

**Table 6:** Species cited by community groups, Group 1 (G1) and Group 2 (G2), with respective use value

Species		Valor de Uso	
Scientific species	Local name	Grupo 1	Grupo 2
BIRDS			
*Crypturellus noctivagus zabele* (Spix, 1825)	zabelê	0.03	0
*Crypturellus parvirostris* (Wagler, 1827)	nambu	0.23	0.28
*Nothura maculosa* (Temminck, 1815)	corduniz	0.33	0.38
*Penelope superciliaris* Temminck, 1815	jacu	0.87	0.85
*Leptotila verreauxi* (Bonaparte, 1855)	juriti	0.32	0.24
MAMMALIA			
*Cabassous unicinctus* (Linnaeus, 1758)	china	0.35	0.21
*Dasypus novencimctus* (Linnaeus, 1758)	tatu comum	0.83	0.76
*Euphractus sexcinctus* (Linnaeus, 1758)	peba	0.82	0.77
*Cerdocyon thous* (Linnaeus, 1766)	raposa	0.01	0.07
*Conepatus semistriatus* (Boddaert, 1785)	gambá	0.02	0.02
*Mazama gouazoubira* (G. Fischer 1814)	veado	1.15	1.49
*Galea spixii* (Wagler, 1831)	preá	0.15	0.16
*Dasypus prymnolopha* (Wagler, 1831)	cutia	0.85	0.84
*Tamandua tetradactyla* (Linnaeus, 1758)	tamanduá	0.08	0.02
REPTILIA			
*Salvator merianae* (Duméril &Bibron, 1839)	teiú	0.05	0.28
MEAN*		0.40	0.42

*Mann-Whitney (U = 30, *p* > 0.05)

Regarding the diversity of known uses by community groups, the Common deer (*M. gouazoubira*) was the only species associated with most categories of established uses, five out of six: meat, used for food (237 citations); lard, leather, horn, hoof, liver and feces as medicinal resources (33 citations); leather and tail for handcraft purposes (28 citations); horn and feet, for magic-religious uses (28 citations); and horn, for other uses (5 citations). We found a high fidelity level (FL) for the use of *M. gouazoubira* for feeding (77.21 %). These results indicate the representative role of the Common deer (*M. gouazoubira*) in the local context, which added to its likely availability in the environment, infers on the local knowledge of this species, which is being passed on in a similar way between the people of the studied groups. Bonifácio et al. [84, 85] studied communities from Chapada do Araripe (Ceará, Brazil) founded that *M. gouazoubira* is recognized as part of the local culture, not only as a food source for survival, but as embedded element to social practice, making this remarkable animal and very popular for local people. In the Bonifácio et al. [85] study thirty-five respondents (100 %) stated that the reason for *M. gouazoubira* hunting in Araripe region in the past and/or present, was (is) the preference for its meat, which confirms that this species is one of the main huntable species of the semiarid region, which its meat is indicated as tasty and smooth for those who hunt it [88].The diversity of uses that *M. gouazoubira* can provide, especially for food and medicine, is a pattern made evident in many etnozoological studies, confirming the great cultural importance of this species as a resource in other rural communities of the neotropical zones [54, 80, 82, 83].

Some studies have shown that people tend to know and use the most abundant species around. Monroy-Vilchis et al. [89], in a study in Mexico with human populations of a protected area observed that the ten species most abundant in the region were the most often used as a source of animal protein. The same was registered for protected area in northeastern Brazil [23].

The multiplicity of uses assigned to natural resources depends on the social group under study and how each individual perceives and uses the environment that lives in [86]. Accordingly, understand the use of animals in cultural perspective becomes a necessary step to promote the maintenance of populations of these species [87]. So we consider that in a management of protected areas should be understood these relationships to avoid conflicts with local culture.

This is because aspects of human behavior are revealed (why use and non-use of an animal, for example) that give meaning and context to the conservation of certain species and therefore should be an aspect to be considered by managers in their management and conservation practices. This is the case of the Araripe National Forest, where the management of fauna requires an understanding of the relationships between people and animals, due to the existence of hunting as part of the local culture of the area.

## Conclusion

Residents from the communities studied who were interviewed, recognize a large number of animal species, with a difference in the knowledge of these species according to the distance of these communities to the FLONA of Araripe, and according to gender.

The inventory-interview analysis of the sampling trace elements method and the use value calculation indicate the Common deer (*M. gouazoubira*) as possibly the most abundant species (common) in this Protected Area, corroborating found by Bonifácio et al. [84, 85]. *M. gouazoubira* is well adapted to semi-arid region and is widely distributed in this region [88].The forest fragments included in Forested Savannah vegetation and the transition area Rainforest/Savannah were the most used environments, both by *M. gouazoubira* and most of the species confirmed by evidence. Thus, actions to ensure the maintenance of the *M. gouazoubira* populations, and to favor the permanence of other species of wildlife negatively affected by the degradation of environments used for feeding and/or reproduction, can contribute to the strengthening of local cultural systems by environmental managers.

Conservation and wildlife management strategies go beyond the endangered species status. Through this study, it is clear that species of interest to local communities and less studied should be included or even have priority in the management of protected areas. This is the case of the *M. gouazoubira* species, the Common deer in the FLONA of Araripe, which has a high cultural value associated with its use versatility. However, the little knowledge we have of their populations puts this species, mistakenly, in the category of less concerning in the lists of endangered species, signaling a greater need to include additional criteria in establishing local conservation strategies.

As a priority measure for the FLONA of Araripe, to direct management efforts, we recommend species monitoring programs, especially those threatened with extinction and also those less studied to assess the short and mid-term trends of these populations in the area of study.

Knowing that the knowledge of the use of natural resources is part of a socio-ecological system that is strongly influenced by the environment, the identification of standards that regulate the relationship between people and animals can also be a conservation strategy for these species and their habitats. Thus, we recommend action plans with programs targeting residents of the National Forest of Araripe at different stages of life, to ensure the continued use of the local fauna, as well as the permanence of the species in protected areas of the Brazilian semi-arid region.

## References

[1] Gomes LP, Rocha CZ, Brandão RA, Marinho Filho J (2015). Mammal richness and diversity in Serra do Facão region, Southeastern Goiás state, central Brazil. Biota Neotropica.

[2] Bezerra DMM, Araujo HFP, Alves RRN (2013). Avifauna de uma área de Caatinga na região do Seridó, Rio Grande do Norte, Brasil. Ornithologia.

[3] Fernandes-Ferreira H, Mendonça SV, Cruz RL, Borges-Nojosa DM, Alves RRN (2013). Hunting of herptofauna in montane, coastal, and drylands áreas of Northeastern Brazil. Herpetological Conserv Biol.

[4] Ferreira FS, Fernandes-Ferreira H, Léo-Neto NA, Brito SV, Alves RRN (2013). The trade of medicinal animals in Brazil: current status and perspectives. Biodivers Conserv.

[5] Licarião MR, Bezerra DMM, Alves RRN (2013). Wild birds as pets in Campina Grande, Paraíba State, Brazil: an ethnozoological approach. An Acad Bras Cienc.

[6] Alves RRN, Rosa II, Léo-Neto NA, Voeks R (2012). Animals for the Gods: magical and religious faunal use and trade in Brazil. Human Ecol.

[7] Alves RRN, Rosa IL (2010). Trade of animals used in Brazilian traditional medicine: trends and implications for conservation. Hum Ecol.

[8] Souto WMS, Mourão JS, Barboza RRD, Mendonça LET, Lucena RFP, ConfessoR MVA, Vieira WLS, Montenegro PFGP, Lopez LCS, Alves RRN (2011). Medicinal animals used ethnoveterinary practices of the Cariri Paraibano, NE Brazil. J J Ethnobiol Ethnomed.

[9] Fernandes-Ferreira H, Mendonça SV, Albano C, Ferreira FS, Alves RRN (2012). Hunting, use and conservation of birds in Northeast Brazil. Biodivers Conserv.

[10] Alves RRN, Leite RC, Souto WM, Bezerra DM, Loures-Ribeiro A (2013). Ethno-ornithology and conservation of wild birds in the semi-arid Caatinga of northeastern Brazil. J Ethnobiol Ethnomed.

[11] Martínez GJ (2013). Use of fauna in the traditional medicine of native Toba (qom) from the Argetina Gran Chaco region: na ethnozoological and conservationist approach. Ethnobiol Conserv.

[12] Santos-Fita D, Naranjo EJ, Rangel-Salazar JL (2012). Wildlife uses and hunting patterns in ruaral communities of the Yucatan Pensinsula, Mexico. J Ethnobiol Ethnomed.

[13] Benítez G (2011). Animals used for medicinal and magico-religious purposes in wetern Granada Province, Andalusia (Spain). J Ethnopharmacol.

[14] Mesquita GP, Barreto LN (2015). Evaluation of mammals hunting in indigenous and rural localities in Eastern Brazilian Amazon. Ethnobiol Conserv.

[15] Bonatto F, Ferreira MN, Figueroa FEV (2009). Efetividade de gestão das unidades de conservação de uso sustentável do estado do Tocantins. Natureza Conservação.

[16] MMA (Ministério do Meio Ambiente) (2007). Áreas Prioritárias para a Conservação, Uso Sustentável e Repartição de Benefícios da Biodiversidade Brasileira: Atualização – Portaria MMA n° 9.

[17] Nascimento JLX, Nascimento ILS, Azevedo Júnior SM (2000). Aves da Chapada do Araripe (Brasil): biologia e conservação. Ararajuba.

[18] Azevedo FR, Moura MAR, Arrais MSB, Nere DR (2011). Composição da entomofauna da Floresta Nacional do Araripe em diferentes vegetações e estações do ano. Rev Ceres.

[19] Ribeiro SC, Roberto IJ, Sales DL, Ávila RW, Almeida WO (2012). Amphibians and reptiles from the Araripe bioregion, northeastern Brazil. Salamandra.

[20] Ribeiro SC, Roberto IJ, Oliveira HF, Oliveira RH, Silva MC, Almeida WO, Avila RW, Albuquerque UP, Meiado MV (2015). Herptofauna da Chapada do Araripe: composição, distribuição e conservação. Sociobidiversidade na Chapada do Araripe.

[21] Cruz MAM, Campello MLC (1998). Conhecendo o Araripe: mastofauna terrestre. Projeto de proteção ambiental e desenvolvimento sustentável da APA Chapada do Araripe e da Biorregião do Araripe.

[22] Ferreira JMR, Thel TN, Tixeira PH, Paula AS, Lyra –Neves RM, Azevedo Júnior SM, Pereira GA, Telino Júniro WR, Albuquerque UP, Meiado MV (2015). Biodiversidade das aves da Chapada do Araripe. Sociobidiversidade na Chapada do Araripe.

[23] Melo RS, Silva OC, Souto A, Alves RRN, Schiel N (2014). The role of mammals in local communities living in conservation areas in the Northeast of Brazil: an ethnozoological approach. Trop Conserv Sci.

[24] CNUC (Cadastro Nacional de Unidades de Conservação). Departamento de Áreas Protegidas. Unidades de conservação por bioma. Disponível em: http://www.mma.gov.br/images/arquivos/areas_protegidas/cnuc/tabela_ucs_bioma_%2012junho2012.pdf. Acessado em: 22 de julho de 2012.

[25] IBAMA (Instituto Brasileiro de Meio Ambiente e Recursos Naturais Renováveis). Plano de Manejo da Floresta Nacional do Araripe, Estado do Ceará. Crato: MMA; 2004. p. 72.

[26] Ribeiro-Silva S, Medeiros MB, Gomes BM, Seixas EM, Silva MAP (2012). Angiosperms from the Araripe National Forest, Ceará, Brazil. Check List.

[27] Lima MF, Lima FAM, Teixeira MMS (1984). Mapeamento e demarcação definitica da Floresta Nacional Araripe – Ceará, Brasil. Ciências Agronômicas.

[28] FUCEME (Fundação Cearense de Meteorologia). Zoneamento Geoambiental do estado do Ceará: parte II mesorregião do sul cearense. Fortaleza: FUCEME; 2006. p. 132.

[29] MMA (Ministério do Meio Ambiente). Espécies ameaçadas 2014. Aves -* Antilophia Bokermanni* Coelho & Silva, 1998 - soldadinho-do-araripe. Disponível em: [http://www.icmbio.gov.br/portal/images/stories/biodiversidade/fauna-brasileira/avaliacao-do-risco/PORTARIA_N%C2%BA_444_DE_17_DE_DEZEMBRO_DE_2014.pdf]. Acessado em: 25 de dezembro de 2014.

[30] MMA (Ministério do Meio Ambiente). Espécies ameaçadas 2014. Aves - *Procnias albus* wallacei Oren & Novaes, 1985 – araponga-da-amazônia. Disponível em: [http://www.icmbio.gov.br/portal/images/stories/biodiversidade/fauna-brasileira/avaliacao-do-risco/PORTARIA_N%C2%BA_444_DE_17_DE_DEZEMBRO_DE_2014.pdf]. Acesso em: 25 de dezembro de 2014.

[31] Albuquerque UP, Ramos MA, Lucena RFP, Alencar NL, Albuquerque UP, Cunha LVFC, Lucena RFP, Alves RRN (2014). Methods and techniques used to collect ethnobiological data. Methods and techniques in Ethnobiology and Ethnoecology.

[32] Bernard HR (1996). Research methods in anthropology: Qualitative and quantitative approaches. Am J Eval.

[33] Phillips O, Gentry AH (1993). The useful plants of tambopata, Peru: I. statistical hypotheses tests with a new quantitative technique. Econ Botany.

[34] Bérnils RS, Costa HC: Répteis brasileiros – Lista de espécies. Version 2012.1. Sociedade Brasileira de Herpetologia. Disponível em: [www.sbherpetologia.org.br.] Acessado em: 15 de outubro de 2012.

[35] CBRO (Comitê Brasileiro de Registros Ornitológicos): Lista das aves do Brasil 2011. Disponível em: [http://www.taxeus.com.br/listamaisinformacoes/122.]. Acessado em: 15 de janeiro de 2012.

[36] Paglia AP, Fonseca GAB, Ryland AB, Herrmann G, Aguiar LMS, Chiarello AG, Leite YLR, Costa LP, Siciliano S, Kierulff MCM, Mendes SL, Tavares VC, Mittermeier RA, Patton JL (2012). Lista anotada dos mamíferos do Brasil.

[37] MMA (Ministério do Meio Ambiente). Lista de Espécies ameaçadas 2014. Disponível em: [http://www.mma.gov.br/biodiversidade/especies-ameacadas-de-extincao/atualizacao-das-listas-de-especies-ameacadas]. Acessado em: 17 de Janeiro de 2016.

[38] IUCN (União Internacional para a Conservação da Natureza e dos Recursos Naturais). Red List of Threatened Species. Version 2014.1. Disponível em: http://www.iucnredlist.org/. Acesso em: 12 de outubro de 2014.

[39] ColwelL RK, Coddington JA (1999). Estimating terrestrial biodiversity through extrapolation. Philos Trans R Soc B.

[40] Magurran AE (2011). Medindo a diversidade biológica.

[41] Alves RRN, Gonçalves MBR, Vieira WLS: Caça, uso e conservação de vertebrados no semiárido brasileiro. Trop Conserv Sci. 2012a, 5(3): 394-416.

[42] Colwell RK: EstimateS: Statistical estimation of species richness and shared species from samples. Version 8.2. 2009. Disponível em: [http://viceroy.eeb.uconn.edu/EstimateS.] Acessado em: 15 de agosto de 2012.

[43] Becker M, Dalponte CJ (1991). Rastros de mamíferos silvestres brasileiros: um guia de campo.

[44] Rossato SC, Leitão-Filho HF, Begossi A (1999). Ethnobotany of Caiçaras of the Atlantic Forest Coast (Brazil). Econ Botany.

[45] Alves RRN: Relationships between fauna and people and the role of ethnozoology in animal conservation. Ethnobiol Conserv. 2012c, 1:2.

[46] OMS (Organização Mundial de Saúde) (2008). Classificação estatística e internacional de doenças e problemas relacionados à saúde: CID-10.

[47] Friedman J, Yaniv Z, Dafini A, Palewitch D (1986). A preliminar classification of the healing potential of medicinal plants, based on a rational analysis of ethnopharmacological field survey among Bedouins in the Negev Desert, Israel. J Ethnopharmacol.

[48] Alencar JBR (2012). Percepção e uso de “insetos” em duas comunidades rurais no semiárido do Estado da Paraíba. Biofar.

[49] Hanazaki N, Alves RRN, Begossi A (2009). Hunting and use of terrestrial fauna used by Caiçaras from the Atlantic Forest coast (Brazil). J Ethnobiol Ethnomed.

[50] Pereira JPR, Schiavetti A (2010). Conhecimento e usos da fauna cinegética pelos caçadores indígenas “Tupinambá de Olivença” (Bahia). Biota Neotropica.

[51] Gil RAP, Guiascón OGR (2012). Uso de la fauna Silvestre en la comunidade maya de Villa Guadalupe, Capeche, México. Etnobiología.

[52] Souza JB, Alves RRN (2014). Hunting and wildlife use in Atlantic Forest remnant of northeastern Brazil. Trop Conserv Sci.

[53] Bezerra DMMSQ, Araujo HFP, Alves RRN (2011). The use of wild birds by rural communities in the semiarid region of Rio Grande do Norte State, Brazil. Bioremediation Biodivers Bioavailability.

[54] Dantas-Aguiar PR, Barreto RM, Santos-Fita D, Santos EB (2011). Hunting activities and wild fauna use: a profile of Queixo D’Antas community, Campo Formoso, Bahia. Bioremediation Biodivers Bioavailability.

[55] Mendez-Cabrera F, Montiel S (2007). Diagnóstico preliminar de la fauna y flora silvestre utilizada por la población maya de das comunidades costeras de Campeche, México. Universidad y Ciencia.

[56] Ibarra JT, Campo CD, Barreau A, Medinaceli A, Camacho CI, Puri R, Martin GJ (2011). Etnoecología chinanteca: conocimiento, práctica y creencias sobre fauna y cacería en un área de conservación comunitaria de la Chinantla, Oaxaca, México. Etnobiología.

[57] Dougherty JWD (1978). Salience and relativity in classification. Am Ethnol.

[58] Gunatilake HM (1998). The role of rural development in protecting tropical rainforests: evidence from Sri Lanka. J Environ Qual.

[59] Pfeiffer JM, Butz RJ (2005). Assessing cultural and ecological variation in ethnobiological research: the importance of gender. J Ethnobiol.

[60] Araújo HFP, Nishida AK (2007). Conhecimento de pescadores artesanais sobre a composição da avifauna em estuários paraibanos: uma contribuição para a conservação. Sitientibus.

[61] Braga H, Schiavetti A (2013). Attitudes and local ecological knowledge of experts fishermen in relation to conservation and bycatch of sea turtles (reptilia: testudines), Southern Bahia, Brazil. J Ethnobiol Ethnomed.

[62] Castilho LC, Martinez RA, Giné GAF, Ribeiro GC, Schiavetti A (2013). The thin-spined porcupine, *Chaetomys subspinosus* (Rodentia: Erethizontidae), within protected áreas in the Atlantic Forest, Brazil: local knowledge and threats. Trop Conserv Sci.

[63] Negrão MFF, Valladares-Pádua CV (2006). Registros de mamíferos de maior porte na Reserva Florestal do Morro Grande, São Paulo. Biota Neotropica.

[64] Caraballo CF (2009). Patrón de uso de hábitat del guazuncho (*Mazama gouazoupira*, Artiodactyla, Cervidae) durante un ciclo anual, en bosques nativos y exóticos del CentroOeste de Entre Ríos. APRONA Boletim Científico.

[65] Duarte JMB, Vogliotti A, Zanetti ES, Oliveira ML, Tiepolo LM, Rodrigues LF, Almeida LB (2012). Avaliação do Risco de Extinção do Veado-catingueiro *Mazama gouazoubira* G. Fischer [von Waldheim], 1814, no Brasil.. Biodiversidade Brasileira.

[66] Feijó A, Langguth A (2013). Mamíferos de médio e grande porte do Nordeste do Brasil: distribuição e taxonomia, com descrição de novas espécies. Rev Nordestina Biologia.

[67] Dubost G, Henry O (2006). Comparison of diets of the acouchy, agouti and paca, the three largest terrestrial rodents of French Guianan forests. J Trop Ecol.

[68] Silva DCB, Fagundes NCF, Teixeira FB, Penha NEA, Santana LNS, Mendes-Oliveira AC, Lima RR (2013). Anatomical and histological characteristics of teeth in agouti (*Dasyprocta prymnolopha* Wagler, 1831). Pesquisa Veterinária Brasileira.

[69] Avila-Pires TCS (1995). Lizards of Brazilian Amazonia (Reptilia: Squamata). Zoologische Mededelingen.

[70] Mendonça LET, Vieira WLS, Alves RRN (2014). Caatinga Ethnoherpetology: relationships between herpetofauna and people in a semiarid region of northeastern Brazil. Amphibian Reptile Conserv.

[71] Kiefer MC, Sazima I (2002). Diet of juvenile tegu lizard Tupinambis merianae (Teiidae) in southeastern Brazil. Amphib-reptil.

[72] Dixo M, Verdade VK (2006). Herpetofauna de serrapilheira da Reserva Florestal de Morro Grande, Cotia (SP). Biota Neotropica.

[73] Zanella N, Paula A, Guaragni AS, Machado LS (2013). Herpetofauna do Parque Natural Municipal de Sertão, Rio Grande do Sul, Brasil. Biota Neotropica.

[74] Silva JS, El-Deir ACA, Moura GJB, Alves RRN, Albuquerque UP. Traditional ecological knowledge about dietary and reproductive characteristics of Tupinambis merianae and Hoplias malabaricus in Semiarid Northeastern Brazil. Human Ecol. 2014. doi:10.1007/s10745-014-9698-9.

[75] Desbiez ALJ, Medri IM (2010). Density and Habitat Use by Giant Anteaters (*Myrmecophaga tridactyla*) and Southern Tamanduas (*Tamandua tetradactyla*) in the Pantanal Wetland, Brazil. Endenata.

[76] MMA (Ministério do Meio Ambiente). Espécies ameaçadas 2014. Mamíferos - *Leopardus tigrinus* (Schreber, 1775). Disponível em [http://www.icmbio.gov.br/portal/images/stories/biodiversidade/fauna-brasileira/avaliacao-do-risco/PORTARIA_N%C2%BA_444_DE_17_DE_DEZEMBRO_DE_2014.pdf]. Acessado em: 25 de dezembro de 2014.

[77] IUCN (União Internacional para a Conservação da Natureza e dos Recursos Naturais). Red List of Threatened Species. Version 2014.1. *Leopardus tigrinus*. Disponível em [http://www.iucnredlist.org/details/11510/0] Acessado em: 10 de junho de 2014.

[78] Barboza RDS, Lopes F, Souto WMS, Fernandes-Ferreira H, Alves RR (2016). The role of game mammals as bushmeat In the Caatinga, northeast Brazil. Ecol Soc.

[79] Alves RRN (2009). Fauna used in popular medicine in Northeast Brazil. Biodivers Conserv.

[80] Altrichter M (2006). Wildlife in the life of local people of the semi-arid Argentine Chaco. Biodivers Conserv.

[81] Medeiros PM, Albuquerque UP. Padrões de uso de plantas medicinais por populações locais: o que pode estar por trás de nossas decisões? In: Albuquerque UP (Org.). Etnobiologia: bases ecológicas e evolutivas. Recife: NUPEEA; 2013:127–145.

[82] Medeiros PM, Ramos MA, Soldati GT, Albuquerque UP. As abordagens ecológico-evolutivas em etnobiologia: histórico e conceitos. In: Albuquerque UP (Org.). Etnobiologia: bases ecológicas e evolutivas. Recife: NUPEEA; 2013:15–36.

[83] Rocha- Mendes F, Mikich SB, Biaconi GV, Pedro WA (2005). Mamíferos do município de Fênix, Paraná, Brasil: etnozoologia e conservação. Rev Brasileira Zoologia.

[84] Bonifácio KM, Freire EMX, Schiavetti A (2016). Cultural keystone species of fauna as a method for assessing conservation priorities in a Protected Area of the Brazilian semiarid. Biota Neotropica.

[85] Bonifácio KM, Schiavetti A, Freire EMX (2015). Conhecimento ecológico local sobre o veado, *Mazama gouazoubira* (G. Fisher, 1814), por moradores do entorno de uma Área Protegida do semiárido brasileiro. Rev Brasileira Ciências Ambientais.

[86] Reyes-García V, Gallois S. Status social e conhecimento ecológico tradicional. In: Albuquerque UP (Org.). Introdução à etnobiologia. Recife: NUPEEA; 2014:181–188.

[87] Santos CAB, Albuquerque UP, Souto WMS, Alves RRN (2016). Assessing the effects of indigenous migration on zootherapeutic practices in the Semiarid Region of Brazil. PLoS One.

[88] Monroy-Vilchis O, Cabrera L, Suárez P, Zarco-Gozález MM, Rodriguez-Soto C, Urios V (2008). Uso traditional de vertebrados silvestres em la Sierra Nanchititla, México. Interciencia.

[89] Alves RRN, Feijó A, Barboza RRD, Souto WMS, Fernandes-Ferreira H, Cordeiro-Estrela P, Langguth A (2016). Game mammals of the Caatinga bioma. Ethnobiol Conserv.

